# Microbiota-gut-kidney axis in health and renal disease

**DOI:** 10.7150/ijbs.125140

**Published:** 2026-01-01

**Authors:** Ying Jin, Shui-Juan Zhang, Shougang Zhuang, Ping Li, Hua Miao, Ying-Yong Zhao

**Affiliations:** 1 School of Pharmaceutical Sciences, Zhejiang Chinese Medical University, No. 548 Binwen Road, Hangzhou, Zhejiang 310053, China.; 2 Department of Medicine, Rhode Island Hospital and Alpert Medical School, Brown University, 593 Eddy St, Providence, Rhode Island, 02903, USA.; 3 Beijing Key Lab for Immune-Mediated Inflammatory Diseases, Institute of Clinical Medical Science, Department of Nephrology, China-Japan Friendship Hospital, Beijing 100029, China.

**Keywords:** gut microbiota, kidney disease, short-chain fatty acids, probiotics, sodium-glucose transport protein 2 inhibitor, fecal microbiota transplantation

## Abstract

Gut microbiota plays a central role in programming host metabolic function and immune modulation in both health and disease. Microbial dysbiosis leads to an increase in opportunistic pathogens and a reduction in beneficial bacteria, which collectively result in the excessive production of detrimental metabolites, particularly uremic toxins such as indoxyl sulfate and trimethylamine-N-oxide, while concurrently decreasing beneficial metabolites, such as short-chain fatty acids and tryptophan catabolites, including indole-3-aldehyde. The accumulation of harmful metabolites and depletion of protective metabolites contribute to fibrosis progression through various mediators, including the renin-angiotensin system, reactive oxygen species, Toll-like receptor 4, aryl hydrocarbon receptor, inhibitor of kappa B/nuclear factor kappa B, and Kelch-like ECH-associated protein 1/nuclear factor erythroid 2-related factor 2 pathways. This review highlights the pathogenic link between gut microbiota and kidney damage via the gut-kidney axis, encompassing acute kidney injury (AKI) and chronic kidney disease (CKD). Innovative therapeutic strategies, including microbial therapeutics (such as probiotics, prebiotics, and synbiotics), natural products (such as neohesperidin, isoquercitrin, and polysaccharides), and fecal microbiota transplantation, have been proposed to restore microbial balance and improve kidney function. Targeted modulation of the gut microbiota offers a promising strategy for developing novel treatments in AKI, CKD, and the transition from AKI-to-CKD. This approach has the potential to prevent or mitigate these conditions and their complications.

## 1. Introduction

**Figure 1:**
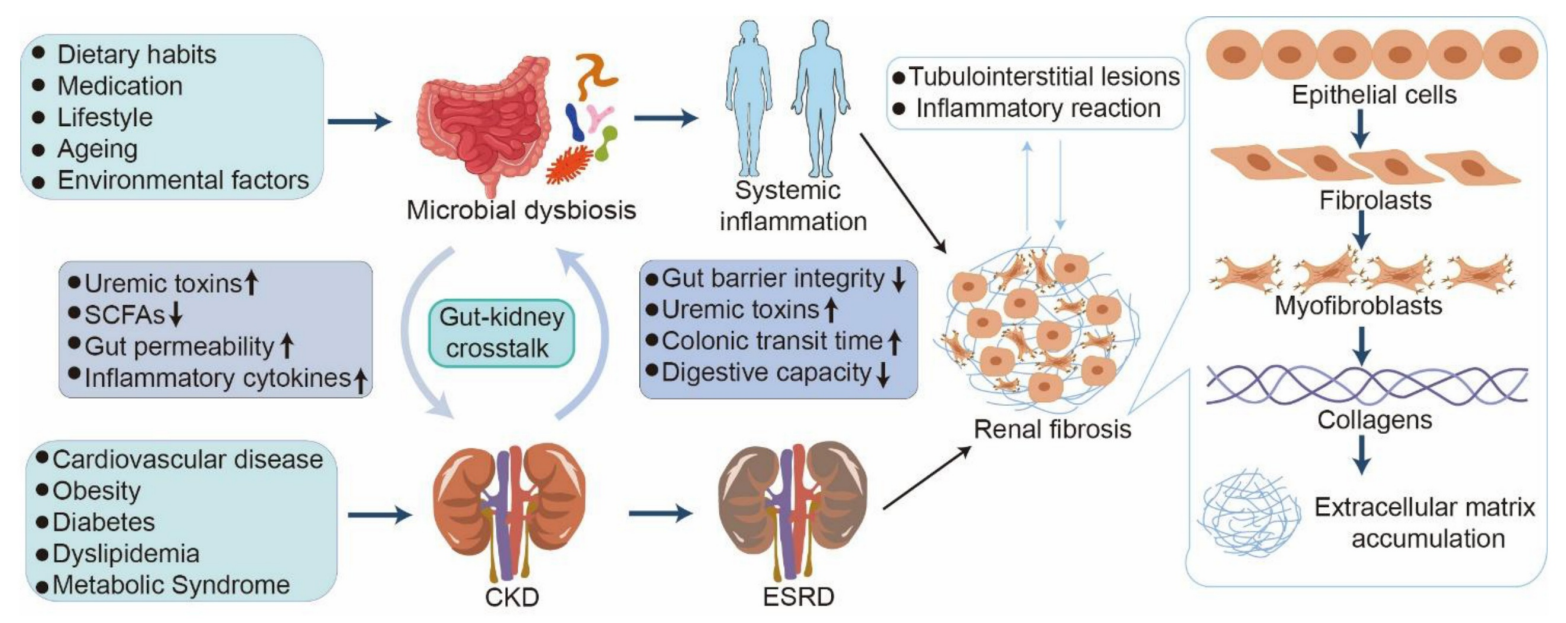
** Crosstalk between microbial dysbiosis and CKD.** CKD induces microbial dysbiosis, which compromises the intestinal mucosal barrier and allows harmful bacteria and uremic toxins to enter the bloodstream. The translocation of these noxious metabolites into circulation can induce oxidative stress and systemic inflammation, subsequently aggravating renal inflammation and fibrosis. During this process, myofibroblasts irreversibly form, followed by the production of multiple types of collagens and accumulation of extracellular matrix, eventually resulting in renal tubulointerstitial fibrosis.

Kidney diseases, including acute kidney injury (AKI) and chronic kidney disease (CKD), are major global public health problems that affect more than 10% of the adult population 1-3. AKI is defined by a rapid elevation in serum creatinine levels, decreasing urine output, or both 4, 5. AKI and CKD are interrelated clinical and pathophysiological disorders 6. AKI can lead to a rapid decline in kidney function and may progress to CKD or end-stage renal disease (ESRD) 7, 8, requiring renal replacement therapies, such as dialysis and transplantation 9, 10. CKD significantly contributes to the burden of non-communicable diseases and correlates with various serious health outcomes, including an elevated risk of mortality, cardiovascular complications, and cognitive decline 11-13. Additionally, kidney disease can occur alongside a variety of other conditions such as cardiovascular disease, obesity, diabetes, dyslipidemia, and metabolic syndrome (**Figure 1**) 14-17.

**Figure 2:**
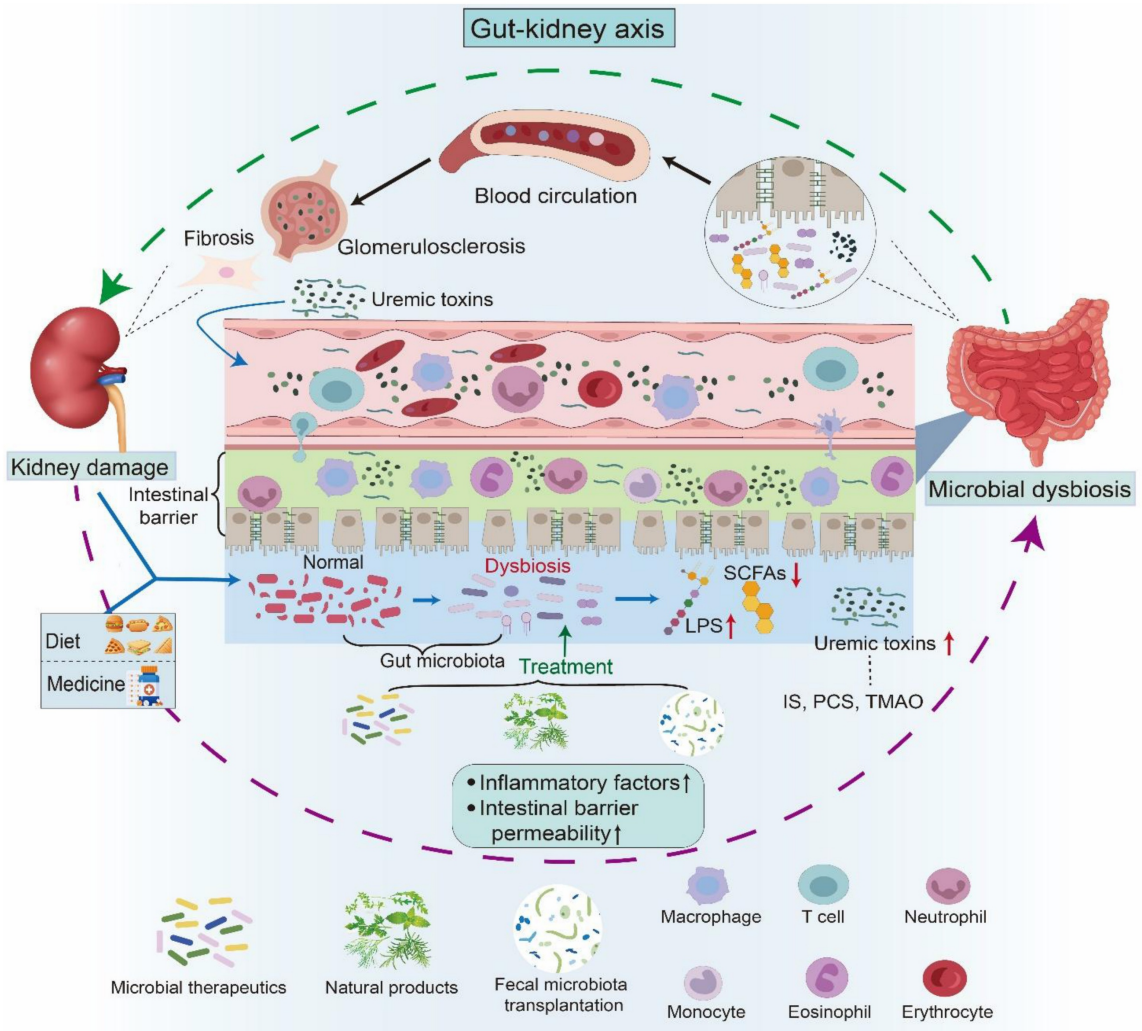
** Gut-kidney axis and its impact on kidney health.** The gut-kidney axis elucidates the relationship between the composition of the gut microbiota and kidney health. Kidney damage, including fibrosis and glomerular sclerosis, can lead to an imbalance in the gut microbiota, thereby affecting the permeability of the intestinal barrier. Concurrently, dietary intake and medication use also contribute to microbial dysbiosis. A disturbance in microbiota composition has been demonstrated to result in heightened levels of uremic toxins, including IS, PCS, and TMAO, along with a decline in LPS and beneficial short-chain fatty acids. Furthermore, compromised intestinal integrity facilitates the entry of uremic toxins into the systemic circulation. These harmful metabolites travel through the bloodstream to the kidneys, activating the corresponding pathways and triggering inflammation and immune responses, leading to kidney damage. Therapies such as microbial therapeutics, fecal microbiota transplantation, and natural products have been shown to influence kidney health by improving the imbalance in gut microbiota.

Irrespective of the initial cause, renal fibrosis, including glomerulosclerosis and tubulointerstitial fibrosis (TIF), represents the terminal outcome of a wide variety of progressive kidney diseases 18, 19. Renal fibrosis is characterized by excessive accumulation and deposition of extracellular matrix components in the kidney parenchyma, leading to tissue scarring (**Figure 2**) 20, 21. Substantial evidence has shown that renal fibrosis is linked to hyperactive pro-fibrotic molecular players, such as the renin-angiotensin system (RAS), reactive oxygen species (ROS), Toll-like receptor 4 (TLR4), and aryl hydrocarbon receptor (AHR) 22-25. Numerous studies have revealed that aberrant pathways, including transforming growth factor-β/Smad (TGF-β/Smad), Wnt/β-catenin, inhibition of kappa B (IκB)/nuclear factor kappa B (NF-κB), and Kelch-like ECH-associated protein 1 (Keap1)/nuclear factor erythroid 2-related factor 2 (Nrf2), are involved in renal fibrosis 23, 26, 27. An increasing number of studies have highlighted that the dysregulation of metabolites, such as amino acids and lipids, including arachidonic acid (AA) and bile acid metabolism, are also implicated in renal fibrosis 28-31. However, the precise molecular mechanisms underlying this pathology remain unclear. Current treatments, including peritoneal dialysis, hemodialysis, colonic dialysis, and kidney transplantation, are costly and non-curative 32. In this context, developing a strategy to target renal fibrosis is essential for effective treatment of patients with kidney disease.

Ample evidence shows dysbiosis of gut microbiota in AKI and CKD, such as diabetic kidney disease (DKD), membranous nephropathy (MN), immunoglobulin A nephropathy (IgAN), focal segmental glomerulosclerosis (FSGS), hypertensive nephropathy (HN), and lupus nephritis (LN), as well as renal replacement therapies including peritoneal dialysis (PD), hemodialysis (HD), and kidney transplantation 24, 33-38. Microbial-derived metabolites, such as short-chain fatty acids (SCFAs), including acetate, propionate, and butyrate, tryptophan derivatives, including indoxyl sulfate (IS), indole-3-aldehyde (IAld), and indole-3-acetic acid (IAA), and lipid derivatives, play a bridging role between the gut and host (**Figure 2**) 39, 40. The accumulation of uremic toxins, such as IS, p-cresol sulfate (PCS), and trimethylamine-N-oxide (TMAO), due to persistent renal injury disrupts the balance of the gut microbiota (**Figure 2**) 39, 41, 42.

This review summarizes the dysbiosis of gut microbiota in kidney disease and elucidates the underlying molecular mechanisms of microbial dysbiosis through a broad spectrum of signaling pathways, such as TGF-β/Smad, IκB/NF-κB, Keap1/Nrf2, phosphatidylinositol-3 kinase (PI3K), and mitogen-activated protein kinases (MAPK), as well as key mediators, such as AHR, NOD-like receptor family pyrin domain containing 3 (NLRP3) inflammasome, and G protein-coupled receptor 43 (GPR43), via alteration of diverse metabolites. Furthermore, targeting the gut microbiota by several therapeutic strategies, such as intestinal microbial therapeutics, natural products, and fecal microbiota transplantation (FMT), are delineated (**Figure 2**). These findings shed light on the molecular mechanisms underlying microbial dysbiosis in kidney disease and provide a clear pathophysiological rationale for treating AKI and CKD. This review provides a more specific concept-driven intervention strategy for developing precise mechanism-based management, prevention, and treatment of kidney disease.

## 2. Gut microbiota in health

The gut microbiota, comprising trillions of microorganisms predominantly inhabiting the gastrointestinal tract, plays a pivotal role in human health and extends beyond digestion 43-45. This critical role encompasses immune modulation, metabolism, and the maintenance of homeostasis 44-47. Gut microbiota homeostasis is influenced by dietary habits, lifestyle, medication use, ageing, and environmental factors (**Figure 1**) 24, 44, 47-49. Meanwhile, changes in the microbial composition adversely affect kidney function, disease progression, and systemic inflammation (**Figure 1**) 24, 35, 50. Gut microbiota plays a pivotal role in metabolic activities, including dietary fiber fermentation, SCFA production, and regulation of gut permeability (**Figure 1**) 51-53. SCFAs, including acetate, propionate, and butyrate, are pivotal for promoting anti-inflammatory responses and influencing systemic metabolism 53-57. Furthermore, the gut microbiota interacts with the host immune system, facilitating immune cell maturation and modulating inflammatory pathways 57-59. A balanced and diverse gut microbiota is integral to overall health, and dysbiosis has been associated with numerous health issues, including kidney diseases (**Figure 1**) 59-62.

Intestinal flora has become a significant factor influencing an individual's overall health, preventing and developing various illnesses, including AKI, CKD, and their complications 63-65. The gastrointestinal microbiome of healthy adults is composed of more than 10^14^ bacteria, of which a variety of bacteria come from four main bacterial phyla, including Firmicutes (79.4%), Bacteroidetes (16.9%), Actinobacteria (2.5%), *Proteobacteria* (1%), and Verrucomicrobia (0.1%) in human feces 66. The Firmicutes phylum, which includes *Lactobacillus* and *Enterococcus,* is responsible for digesting complex carbohydrates and producing anti-inflammatory metabolites such as SCFAs, including butyrate, propionate, and acetate 53, 57. The Bacteroidetes phylum, which includes *Bacteroides* and *Prevotella*, consists of anaerobic Gram-negative bacteria 44, 67. *Bacteroides* serve as intestinal symbionts, forming a protective barrier against pathogens and aiding in the supply of nutrients to other microbiota 67, 68. However, *Prevotella* colonization reduces SCFA production, increases susceptibility to mucosal inflammation, and stimulates immune cells to secrete inflammatory mediators 68, 69. Actinobacteria, represented by *Bifidobacteria*, play a pivotal role in maintaining intestinal health 70. An increase in *Proteobacteria*, including pathogens such as *Escherichia coli* (*E. coli*), is attributed to dysbiosis and exacerbates inflammation or enables the incursion of exogenous pathogens 71. *Akkermansia muciniphila*, a member of the *Verrucomicrobia*, generates anti-inflammatory lipids and enhances the intestinal barrier function 72-74. In summary, the concept of the gut-kidney axis has emerged, highlighting the bidirectional communication between gut microbiota and renal function (**Figure 1**) 75. Given the pivotal role of the gut microbiota in maintaining health and its disruption in kidney disease, it is essential to explore how dysbiosis manifests in specific conditions such as AKI and the subsequent transition to CKD.

## 3. Gut microbial dysbiosis and dysregulation of microbial-derived metabolites in kidney disease

Under homeostatic conditions, the symbiotic interaction between microorganisms and the host facilitates metabolism and exerts diverse health benefits 44, 76, 77. The commensal intestinal microflora stimulates and potentiates host immune response through nutrient competition, thereby hindering pathogens from establishing themselves in the host 44, 53, 78. The intestinal microflora regulates immune, metabolic, and endocrine functions 53, 79, 80. However, drug use and unhealthy diet lead to microbial dysbiosis, triggering metabolic disorders and adversely affecting kidney health (**Figure 2**) 24, 36. This ecological disruption is typically characterized by the reduction of beneficial bacteria such as *Lactobacillus* and *Bifidobacterium*, which play a protective role against pathogenic organisms 24, 36, 81. Concurrently, uncontrolled proliferation of harmful bacteria, such as certain strains of *E. coli*, leads to a shift in microbial composition that disrupts normal metabolic processes 82, 83. This alteration results in the accumulation of toxic metabolites that can adversely affect renal function (**Table 1**) 84, 85. Dysbiosis is also linked to systemic inflammation, a recognized factor in CKD progression 35, 84. Excessive growth of pathogenic bacteria increases pro-inflammatory cytokines, thereby exacerbating inflammatory responses and renal pathological injury 86. For example, uremic toxins such as IS and PCS are generated through the metabolism of dietary proteins from pathogenic bacteria 87. Their accumulation in the bloodstream, particularly when renal function is compromised, contributes to further kidney damage and associated metabolic disorders (**Figure 2**) 87. Moreover, dysbiosis disrupts the metabolic pathways that govern energy homeostasis and nutrient absorption 88. A reduction in the production of SCFAs, particularly butyrate, due to reduced populations of beneficial intestinal bacteria compromises the integrity of the intestinal barrier and promotes inflammation (**Table 1**) 89, 90. These diminished SCFAs lead to increased intestinal permeability, facilitating translocation of bacterial endotoxins into systemic circulation, which further exacerbates inflammation and metabolic dysregulation (**Table 1**) 89, 90.

**Figure 3:**
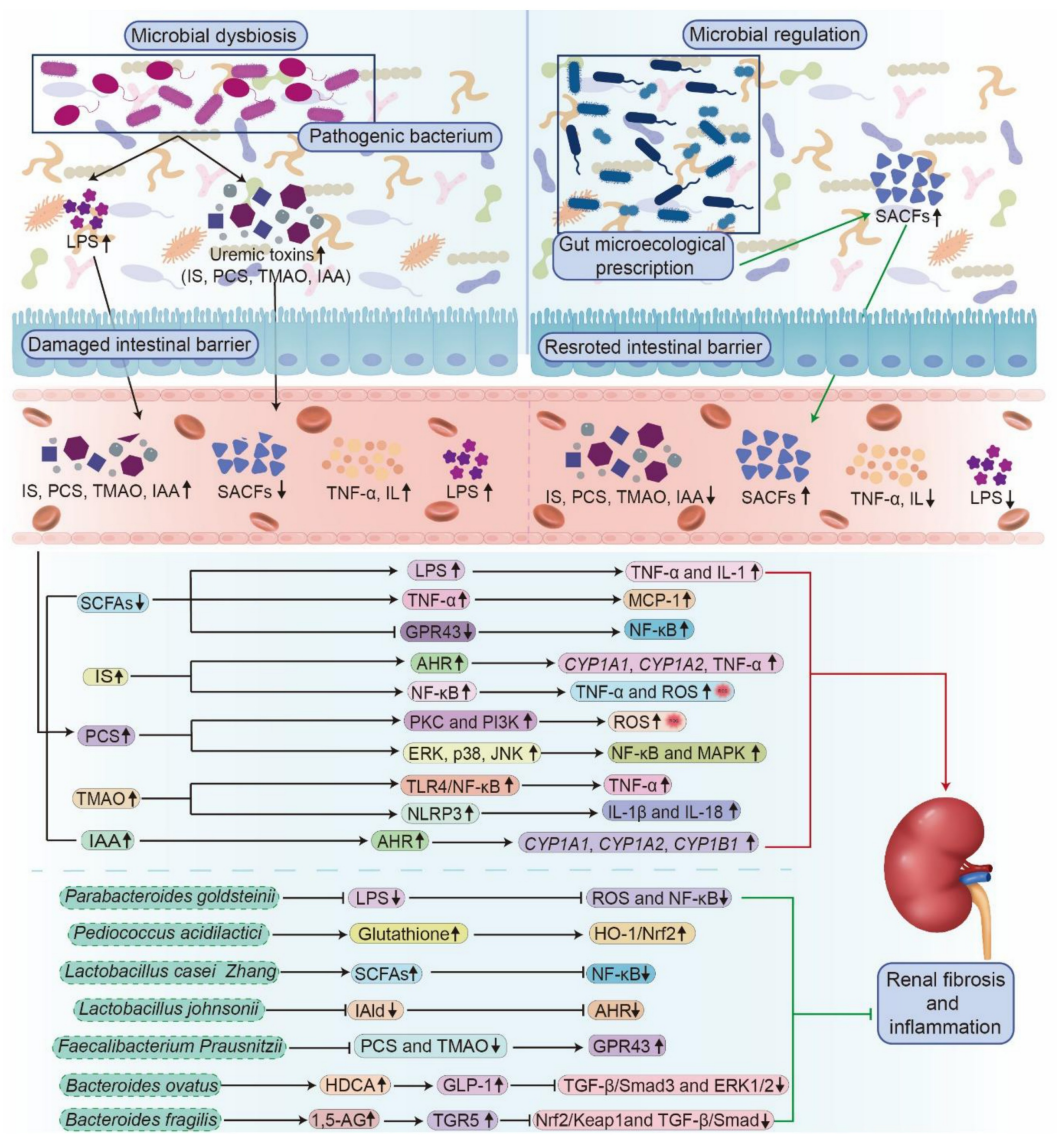
** The molecular mechanism of kidney diseases mediated by microbial dysbiosis and the renal protective effect of gut microecological preparations.** Dysbiosis, characterized by a reduction in beneficial SCFAs and an increase in uremic toxins (IS, PCS, TMAO, and IAA), damages the intestinal barrier and elevates LPS and pro-inflammatory cytokines (TNF-α, IL). These toxins and signals activate multiple inflammatory pathways and oxidative stress, collectively aggravating renal inflammation and fibrosis. Conversely, treatment with specific beneficial bacteria enhances their protective effects, such as producing SCFAs and lowering uremic toxins, leading to the suppression of NF-κB and TGF-β/Smad pathways, reducing oxidative stress, and activating the Nrf2 pathway. Thus, dysbiosis drives kidney damage through toxin release, barrier failure, and inflammation, while gut microecological preparations weaken the key mechanisms that regulate renal inflammation and fibrosis.

Evidence shows that SCFAs mitigate renal fibrosis by suppressing the expression of the pro-fibrotic chemokine monocyte chemoattractant protein-1 (MCP-1) induced by tumor necrosis factor-α (TNF-α) (**Figure 3**) 88. Furthermore, SCFAs activate GPR43, leading to blocking IκB phosphorylation and degradation, which inhibits NF-κB activity and reduces inflammatory gene expression (**Figure 3**) 88. Concurrently, SCFAs strengthen the acetylation state of mitogen-activated protein kinase phosphatase 1 (MKP-1) by inhibiting histone deacetylases (HDACs), which ultimately suppress lipopolysaccharide (LPS)-induced TNF-α, interleukin (IL)-1, and nitrite synthesis while restraining renal tubular cell apoptosis (**Figure 3**) 88. Additionally, an imbalance in intestinal flora also interferes with the metabolism of key nutrients, such as proteins, lipids, and carbohydrates, thereby influencing kidney health 91, 92. Dysbiosis leads to impaired lipid metabolism, resulting in dyslipidemia, a common complication among patients with kidney disease that precipitates atherosclerosis and cardiovascular disorders 93. Furthermore, dysbiosis worsens insulin resistance, complicating the management of metabolic disorders in patients with kidney disease 94, 95. Taken together, gut microbial dysbiosis exerts a critical influence on individuals with kidney disease.

### 3.1 AKI

AKI is characterized by a rapid decline in renal function and is typically diagnosed by elevated serum creatinine levels and reduced urine output 4. Diverse etiologies of AKI, including drug toxicity, ischemia-reperfusion injury (IRI), and sepsis, are associated with distinct alterations in gut microbiota composition 96-99. Cisplatin is one of the most common causes of drug-induced AKI 96. In animal models, cisplatin-induced AKI has been associated with an increased abundance of *Allobaculum*, *Lactobacillus*, *Alloprevotella*, and *Bacteroides* 96. Similarly, Yang et al. reported that cisplatin-treated mice exhibited increased relative abundances of *Dubosiella*, *Escherichia-Shigella*, and *Ruminococcus*, along with decreased abundances of *Ligilactobacillus* and *Anaerotruncus* 97.

IRI has drawn considerable attention in experimental models. Compared with sham-operated controls, IRI mice demonstrated a decreased abundance of *Bifidobacterium* and increased abundances of *Lactobacillus*, *Clostridium*, and *Ruminococcus* 98. In renal IRI-induced AKI models, the relative abundance of *Parabacteroides goldsteinii (P. goldsteinii)* was elevated, consistent with findings in patients with AKI 100. Moreover, in sepsis-associated AKI, the accumulation of metabolic waste products caused by impaired renal excretion contributes to gut microbial dysbiosis 99. In patients with sepsis, increased abundances of Enterobacteriaceae and Lachnospiraceae have been identified as predictors of subsequent AKI onset 100. Winner et al. further reported that in a murine model of sepsis, elevated serum creatinine levels were accompanied by an increased relative abundance of Lachnospiraceae spp. 101. During AKI, elevated inflammatory cytokines and ischemia alter the composition of the intestinal microbiota, leading to reduced microbial diversity and compromised intestinal barrier integrity 102. Li et al. found that the relative abundances of *Streptococcus*, *Escherichia*, *Pseudoflavonifractor*, *Rothia*, *Granulicatella*, *Peptostreptococcus*, and *Actinomyces* increased in AKI patients, whereas *Gemmiger, Erysipelatoclostridium*, *Coprococcus*, and *Ruminococcus* decreased^103^. Microbial dysbiosis not only reduces SCFA levels, thereby aggravating inflammation and kidney injury, but also enhances the production of uremic toxins, accelerating renal deterioration (**Figure 2**) 102, 104. In summary, AKI and gut microbial dysbiosis form a vicious cycle; renal injury promotes dysbiosis, which in turn exacerbates inflammation, oxidative stress, and toxin accumulation, thereby intensifying kidney damage and accelerating disease progression (**Figure 2**) 104.

### 3.2. Chronic renal injury

#### 3.2.1. CKD

CKD is characterized by irreversible pathological injury that ultimately progresses to ESRD 105. In a comparative study of healthy individuals and patients across five stages of CKD, the taxonomic chain Bacilli, Lactobacillales, Lactobacillaceae, *Lactobacillus,* and *Lactobacillus johnsonii* (*L. johnsonii*) was found to correlate with CKD progression. The abundance of *L. johnsonii* was associated with the estimated glomerular filtration rate and clinical markers of kidney function, including serum creatinine, urea, and cystatin C levels 106. The serum concentration of IAld, a tryptophan catabolite produced by *L. johnsonii*, is decreased in late-stage CKD patients and in CKD rats induced with 5/6 nephrectomy (NX) or unilateral ureteral obstruction (UUO), showing a negative correlation with serum creatinine (**Table 1**) 106. IAld treatment ameliorated renal injury by inhibiting AHR pathway in rats with renal fibrosis and 1-hydroxypyrene-stimulated HK-2 cells. The protective effect of IAld was partially abrogated in AHR-deficient mice and HK-2 cells (**Table 1**) 106. These findings provide mechanistic insights into how tryptophan metabolism mediated by gut microbiota influences CKD progression and identify a potential therapeutic target for intervention.

CKD patients also exhibit a decreased relative abundance of *Faecalibacterium* 107. Several studies have reported reductions in *Bacteroides fragilis* (*B. fragilis*) and *Bacteroides ovatus* (*B. ovatus*) in CKD patients and UUO mice 108, 109. The relative abundance of *Clostridium scindens* (*C. scindens*) was also decreased in UUO models 108. Moreover, ESRD patients showed increased abundance of *Eggerthella lenta*, *Flavonifractor plautii*, *Alistipes finegoldii*, *Alistipes shahii*, *Ruminococcus*, and *Fusobacterium*, along with decreased abundance of *Prevotella copri*, *Clostridium*, and several butyrate-producing bacteria such as *Roseburia*, *Faecalibacterium prausnitzii*, and *Eubacterium rectale* 110. These compositional shifts are accompanied by elevated levels of uremic toxins (IS, PCS, indole, and p-cresol) and secondary bile acids derived from aromatic amino acid degradation and microbial secondary bile acid biosynthesis (**Table 1**) 110. The decline in fecal SCFAs, including acetate, propionate, and butyrate, correlates with the reduction in SCFA-producing species in ESRD patients (**Table 1**) 110. SCFAs, including acetate, propionate, and butyrate, exert protective effects on kidney diseases through the modulation of inflammation and oxidative stress, primarily via the activation of G protein-coupled receptors, leading to reduced pro-inflammatory cytokine production, lowered oxidative stress markers, thereby improved renal function (**Table 1**) 89. Therefore, reducing the number of bacteria that produce SCFAs disrupts the balance of metabolites produced by the intestinal microbiota, affecting their impact on the kidneys. For example, insufficient production of the protective compound butyrate reduces its anti-inflammatory and anti-fibrotic effects by inhibiting NF-κB pathway 89. Concurrently, alterations in the gut microbiota impact uremic toxin metabolism, as certain butyrate-producing bacteria themselves contribute to indole sulfate production, which in turn exacerbates renal injury (**Table 1**) 84, 89. These findings highlight the dysbiosis of the gut microbiota and dysregulation of microbial metabolites in CKD (**Figure 2**).

**Figure 4:**
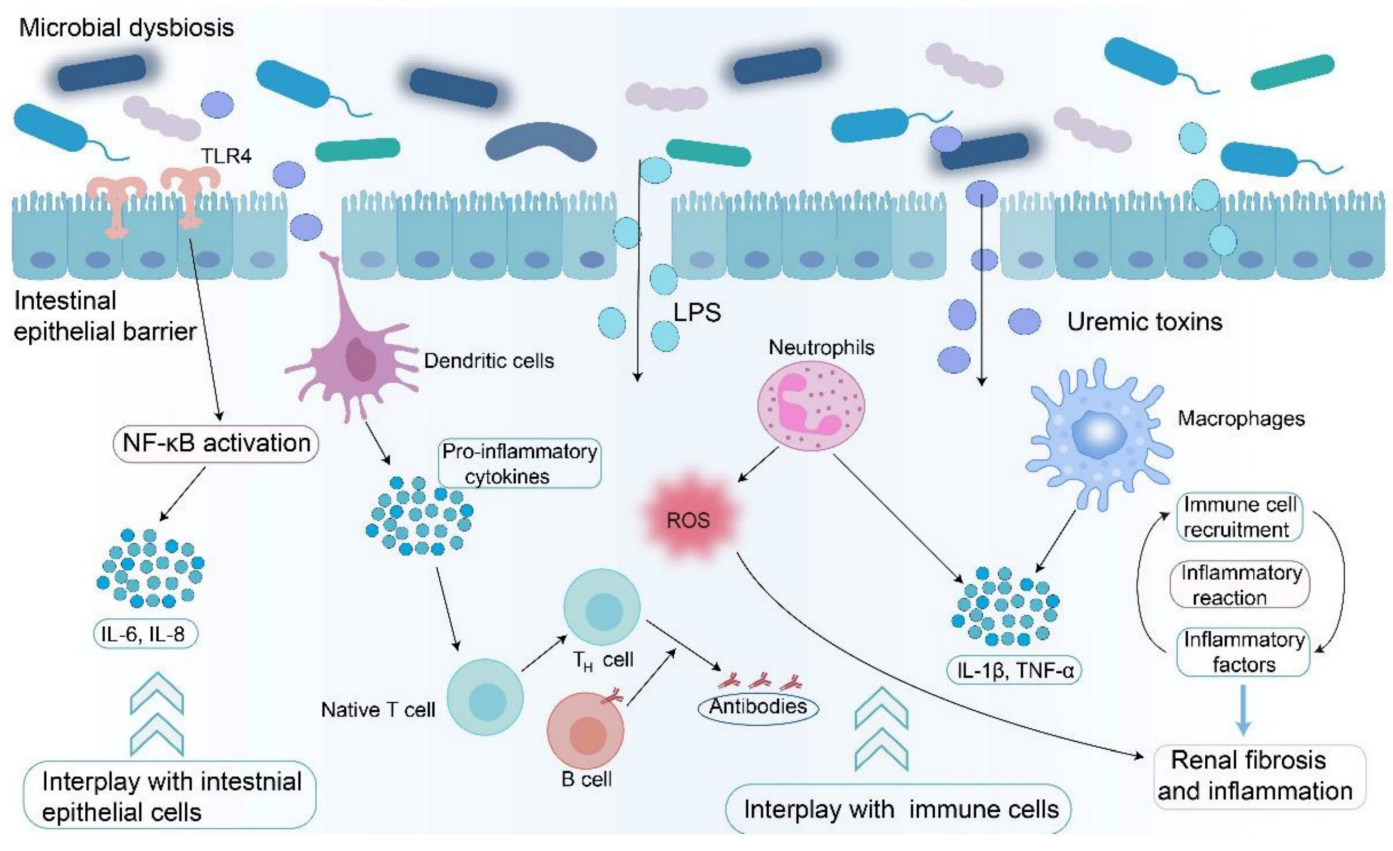
** Impact of microbial dysbiosis on inflammatory response and immune system.** Microbial dysbiosis leads to the accumulation of uremic toxins and LPS, which affect the immune system and inflammatory response via two pathways. Harmful metabolites interact with intestinal epithelial cells, leading to activation of NF-κB signaling pathway. Harmful metabolites cross the gut intestinal barrier, activate immune cells, including dendritic cells, macrophages, and neutrophils, and produce pro-inflammatory cytokines, such as IL-1β and TNF-α, leading to inflammatory reactions. Harmful metabolites also promote antigen presentation and initiate host adaptive immune responses by affecting the inflammatory factors. Microbial dysbiosis exacerbates renal fibrosis by increasing the levels of uremic toxins and LPS and by regulating the immune system and inflammatory response.

CKD disrupts the gut microbial equilibrium, establishing a feedback loop that exacerbates disease progression (**Figure 2**) 24, 33, 65. Dysbiosis leads to increased levels of uremic toxins and decreased SCFAs (**Figure 2**) 24, 84. This dysbiosis is driven by multiple factors, including intestinal urea accumulation, impaired ammonia metabolism secondary to renal dysfunction, dietary modifications, and medical interventions (**Figure 2**) 35, 111. The deleterious effects of dysbiosis manifest through three major mechanisms. Impairment of the intestinal barrier facilitates the translocation of bacteria and microbial metabolites, such as IS and LPS, into systemic circulation, inducing endotoxemia (**Figure 2**) 112. Dysbiosis increases the production of harmful metabolites such as TMAO, which promotes vascular injury and increases cardiovascular risk 42. Uremic toxins and pathogen-associated molecular patterns activate the immune system and sustain systemic inflammation (**Figure 4**) 34. This complex interplay within the gut-kidney axis not only accelerates renal functional decline, but also diminishes patients' quality of life by altering nutritional metabolism and increasing the incidence of cardiovascular complications 42, 75. Experimental evidence supports the pathological impact of dysbiosis. Hyperuricemia-induced microbial dysbiosis increases L-kynurenine levels, which activates AHR pathway in renal tissue and promotes fibrosis 113. Animal studies suggested that beneficial metabolites such as butyrate have been shown to lower blood pressure and reduce inflammation by suppressing the renin-angiotensin system in animal studies 114.

At the cellular level, *in vitro* studies have revealed that microbial metabolites directly influence kidney cell viability and inflammatory signaling. IS induces cytotoxicity in renal tubular cells by upregulating arachidonate 12-lipoxygenase expression and increasing 12(S)-hydroxyeicosatetraenoic acid production 115. IS also enhances the expression of cytochrome P450 family 1 subfamily A member 1 (*CYP1A1*), cytochrome P450 family 1 subfamily B member 1 (*CYP1B1*), and inflammatory cytokines (TNF-α) in T cells through activating AHR pathway, as well as modulating inflammatory responses and cell cycle regulation (**Figure 3**) 84. Furthermore, IS activates NF-κB signaling, increasing pro-inflammatory factors such as cyclooxygenase-2, ROS, TNF-α, IL-6, and IL-1β, while suppressing antioxidant factors such as heme oxygenase-1 and superoxide dismutase 2, through Nrf2 pathway inhibition, thereby disrupting intestinal homeostasis and promoting systemic inflammation in mice (**Figure 3**) 116. Similarly, PCS enhances ROS generation via the protein kinase C and PI3K pathways, leading to elevated inflammatory cytokine expression and renal tubular cell injury (**Table 1** and **Figure 3**) 84. PCS also induces apoptosis and autophagy by activating NF-κB and MAPK signaling and increasing the phosphorylation of extracellular signal-regulated kinase (ERK), p38, and c-Jun N-terminal kinase (JNK) in HK-2 cells (**Figure 3**) 117. TMAO further exacerbates renal damage and calcium oxalate deposition by inducing oxidative stress, autophagy, and apoptosis in renal cells (**Figure 3**) 118. In summary, CKD-related gut microbial dysbiosis accelerates renal function decline by sustaining a vicious gut-kidney axis cycle that amplifies oxidative stress, inflammation, and metabolic toxicity (**Figure 3**).

#### 3.2.2. DKD

DKD is one of the most prevalent complications of diabetes mellitus and the leading cause of ESRD worldwide 119, 120. Emerging evidence indicates that alterations in the gut microbiota composition are closely associated with DKD, and microbial dysbiosis contributes to disease progression 121, 122. Patients with DKD exhibit a decreased Firmicutes-to-Bacteroidetes ratio and increased relative abundance of *Clostridium* 122. Another study reported elevated relative abundances of *Selenomonadales*, *Neosynechococcus*, *Shigella*, *Bilophila*, *Acidaminococcus*, and *Escherichia* in patients with DKD, whereas non-DKD individuals displayed higher relative abundance of *Phlyctochytrium* 123. Further analysis revealed that the relative abundances of *Citrobacter farmeri* and *Syntrophaceticus schinkii* were positively correlated with the urinary albumin-to-creatinine ratio in patients with DKD 123. These microbial alterations are thought to trigger inflammatory and immune responses by regulating LPS biosynthesis, SCFA production, and carbohydrate metabolism, thereby exacerbating DKD progression (**Figure 4**) 123. Du et al. identified increased relative abundances of *Megasphaera*, *Veillonella*, *Escherichia-Shigella*, *Anaerostipes*, and *Haemophilus* as potential microbial indicators of DKD 124. Similarly, Chen et al. reported that the relative abundances of *Oscillibacter*, *Bilophila*, UBA1819, *Ruminococcaceae* UCG-004, *Anaerotruncus*, and *Ruminococcaceae* NK4A214 increased in DKD patients compared with non-DKD controls 125. The overgrowth of pathogenic bacteria not only amplifies host inflammatory responses but also accelerates disease progression 125. Zhang et al. further demonstrated that the relative abundances of butyrate-producing bacteria, including *Clostridium*, *Eubacterium*, and *Roseburia intestinalis*, as well as potential probiotics such as *Lachnospira* and *Intestinibacter*, were decreased in DKD patients, while *Bacteroides stercoris* abundance was increased 126. These microbial changes correlated with clinical indicators of lipid metabolism, glucose metabolism, and renal function 126. Dysbiosis-induced disruption of the intestinal barrier aggravates DKD by activating inflammatory responses and reducing SCFA levels (**Figure 2** and** Figure 4**) 126.

Experimental studies using animal models have corroborated these findings. The relative abundances of *CAG-352 and Ruminococcus_sp_YE281* were increased in DKD rats, whereas the abundance of *Anaerobiospirillum* was decreased compared to that in normal controls 127. In streptozotocin (STZ)-induced diabetic mice, the relative abundance of *Lachnospiraceae_NK4A136_group* and *norank_f_Muribaculaceae* was reduced, while the abundance of *Dubosiella* increased 128. Mechanistically, microbial dysbiosis exacerbates renal injury and fibrosis by increasing intestinal permeability, disrupting lipid metabolism, inducing ROS-mediated oxidative stress, and activating TLR4-mediated inflammatory responses (**Figure 4**) 128. Furthermore, animal models of DKD show a reduced abundance of beneficial bacteria such as *Bifidobacterium* and *Prevotella*, along with increased abundance of *Escherichia-Shigella* 127, 129. Although animal models cannot fully replicate human diseases, these findings emphasize the essential role of intestinal flora in the development and progression of DKD. The intestinal microbiota and its metabolites, particularly SCFAs, play a pivotal role in the pathogenesis of DKD 130. Microbial dysbiosis induces oxidative stress, leading to excessive ROS generation, which subsequently activates the NLRP3 inflammasome 92. This activation triggers inflammation in glomerular endothelial cells and exacerbates renal dysfunction in DKD 92. Taken together, microbial dysbiosis, activation of RAS, oxidative stress, and inflammation form a complex, interdependent pathophysiological network that drives the onset and progression of DKD.

#### 3.2.3. MN

MN is one of the most common causes of adult nephrotic syndrome and is characterized by the deposition of immune complexes beneath the glomerular basement membrane 40, 131, 132. Both cationic bovine serum albumin (CBSA)-induced MN rats and idiopathic membranous nephropathy (IMN) patients exhibit a decreased relative abundance of *L. johnsonii*, *Lactobacillus reuteri*, *Lactobacillus vaginalis*, *Lactobacillus murinus*, and *Bifidobacterium animalis* in feces, along with altered serum levels of indole-3-pyruvic acid, IAld, tryptamine, indole-3-lactic acid, and IAA 133. Further analyses revealed that both CBSA-induced MN rats and IMN patients displayed increased mRNA expression of AHR and its downstream genes *CYP1A1*, *CYP1A2*, and *CYP1B1*, accompanied by decreased cytoplasmic AHR and increased nuclear AHR protein expression in renal tissue (**Figure 3**) 132. Additionally, fecal samples from MN patients demonstrated an increased relative abundance of Proteobacteria, Actinobacteria, *Escherichia*-*Shigella*, *Subdoligranulum*, and *Bifidobacterium*, together with decreased abundances of Bacteroidota, *Bacteroides*, *Prevotella*, and *Megamonas*, which were associated with MN onset 134. The relative abundance of *Citrobacter* and *Akkermansia* in feces was correlated with clinical serum parameters in patients with MN 135. These microbial alterations were accompanied by significant changes in the fecal tryptophan metabolism 135.

FMT experiments further demonstrated that gut microbial dysbiosis enhances the intestinal permeability in microbiota-depleted mice and mediates intrarenal activation of NOD-like receptor signaling 135. Moreover, MN rats exhibited colonic structural injury, an increased Firmicutes-to-Bacteroidetes ratio, an elevated relative abundance of *Allobaculum* and *Desulfovibrio*, greater production of uremic toxin precursors, and reduced levels of SCFAs 136. These findings deepen our understanding of the intricate interactions between MN and the gut microbiome, providing valuable insights and identifying potential microbial targets for future therapeutic strategies for MN management.

#### 3.2.4. IgAN

IgAN is the most prevalent form of primary glomerulonephritis and has become a major cause of CKD worldwide 131, 137. A hallmark of IgAN is the accumulation of IgA-containing immune complexes in the glomerular mesangium. IgA, which is predominantly synthesized in the intestinal mucosa, serves as a critical barrier against bacterial pathogens. Alterations in the gut microbiome and its metabolites have been identified as potential factors influencing disease susceptibility and progression 138, 139. Patients with IgAN exhibit an increased relative abundance of Firmicutes, particularly Ruminococcaceae and Lachnospiraceae, along with a reduced abundance of beneficial bacteria, such as *Clostridium* and *Lactobacillus* 139. Furthermore, patients with IgAN display an expansion of the taxonomic chain Proteobacteria-Gammaproteobacteria-Enterobacteriales-Enterobacteriaceae-*Escherichia-Shigella*, which is reversed following immunosuppressive therapy 140. This microbial pattern is associated with pathways promoting infectious diseases and xenobiotic metabolism 140. Among these taxa, *Escherichia-Shigella* contributes most significantly to the optimal bacterial classifier for distinguishing IgAN 140.

**Figure 5:**
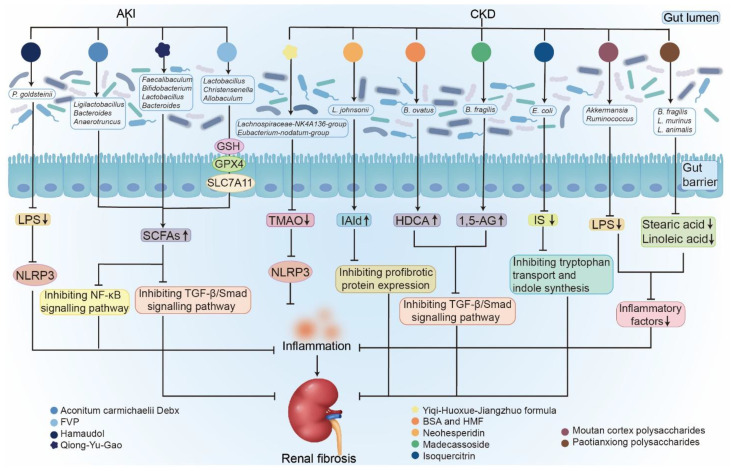
** Comprehensive effects of natural products on kidney diseases by regulating gut microbiota.** Natural products, including TCMs and plant extracts, are important therapies for intervening in diverse kidney diseases, including AKI and CKD. They retard renal fibrosis in a gut microbiome-dependent pathway. They attenuated AKI by increasing levels of SCFAs and decreasing levels of LPS, inhibiting NF-κB and TGF-β/Smad signaling pathways, and upregulating GSH, GPX4, and SLC7A11 expression. They improved renal fibrosis and inflammation by increasing levels of SCFAs and IAld, decreasing levels of uremic toxins, inhibiting TGF-β/Smad signaling pathway, as well as inflammatory factors, reducing tryptophan transport and indole synthesis.

Elevated serum levels of specific metabolites, such as B-cell activating factor, along with increased intestinal permeability and inflammation, further suggest the link between microbial dysbiosis and immune dysregulation 139, 140 (**Figure 5**). These alterations promote the production of nephrotoxic galactose-deficient IgA1 and exacerbate disease progression 139, 140. Notably, immunosuppressive therapy not only reduces the proliferation of pathogenic bacteria but also restores gut microbial balance and improves clinical outcomes 139. In addition, IgAN patients show a decreased relative abundance of *Bifidobacterium*, which negatively correlates with proteinuria and hematuria levels, suggesting that a reduction in *Bifidobacterium* abundance is associated with IgAN severity 141. Probiotic treatment containing *Bifidobacterium* in IgAN mice alleviates microbial dysbiosis by increasing the abundance of beneficial bacteria and reducing the abundance of pathogenic bacteria. Mechanistically, both probiotics and SCFAs ameliorate IgAN manifestations by suppressing the NLRP3/ASC/Caspase-1 signaling pathway 141.

Several studies in IgAN animal models have reported a decreased abundance of beneficial bacteria, including *Bifidobacterium*, *Oscillospira*, *Roseburia*, *Turicibacter*, and *Dubosiella* 141-143, along with increased levels of pathogenic bacteria, such as *Helicobacter*, *Alloprevotella*, *Shigella sonnei*, *Streptococcus danieliae*, *Desulfovibrio fairfieldensis*, and *Candidatus Arthromitus* 141, 142. These alterations are accompanied by diminished microbial diversity indices and abnormal metabolic profiles, including decreased SCFA and polyunsaturated fatty acid levels, indicating a compromised intestinal metabolic state 142. Studies indicated that microbial dysbiosis in IgAN disrupts intestinal barrier integrity and metabolic balance, leading to the proliferation of pathogenic bacteria and a reduction in beneficial metabolism, thereby upsetting systemic immune homeostasis 142, 143. This process promotes the formation and deposition of IgA-containing immune complexes in the glomerular mesangial region, thereby triggering inflammation and injury 143. Furthermore, immunosuppressive therapy improves renal lesions, reverses dysbiosis, and suppresses related inflammatory pathways, revealing the intricate interactions within the microbiome-gut-kidney axis 139. In summary, microbial dysbiosis is a potential pathogenic factor in IgAN, inducing intestinal dysfunction and triggering systemic immune dysregulation to ultimately cause renal injury.

#### 3.2.5. FSGS

FSGS is a common morphological manifestation of nephrotic, characterized primarily by podocyte injury 144, 145. Increasing evidence indicates that patients with FSGS exhibit gut microbial dysbiosis 146. In animal models of nephrotic syndrome, a decrease in gut microbial diversity has been observed, accompanied by a compositional shift from Firmicutes to Bacteroidetes and alterations in specific bacterial taxa that increase the production of precursors for harmful metabolites and uremic toxins 147. Shi et al. reported that in rats with FSGS, the relative abundances of *Bifidobacterium*, *Collinsella*, *Paludibacter*, *Subdoligranulum*, *Megamonas*, and *Coprobacter* increased, whereas *Granulicatella* and *Christensenella* decreased 148. In addition, in the Adriamycin-induced FSGS model rats, the relative abundances of *Lachnospiraceae*_*NK4A136_group* and *Prevotellaceae*_*UCG-001* were reduced, while *[Ruminococcus]_torques_group* abundance was increased 149. Further animal studies have demonstrated that restoring the microbial balance in FSGS rats suppresses pathogenic bacterial proliferation and alleviates renal injury 148. These findings suggest that the gut microbiota and its metabolites play critical roles in the pathogenesis of FSGS and represent promising targets for future therapeutic interventions.

#### 3.2.6. HN

Hypertension is widely recognized as a critical factor in the pathogenesis of CKD 38. As a vital endocrine and metabolic organ, the kidney is highly susceptible to hypertensive injury, which contributes to progressive renal dysfunction 150. Given the complex interactions between the gut microbiome and systemic physiology, dysbiosis, characterized by altered microbial composition and reduced diversity, plays a significant role in the pathogenesis of HN 151. Wang et al. reported an increased relative abundance of *Klebsiella*, *Turicibacter*, and *Enterobacte*r in patients with HN, accompanied by a decreased abundance of *Blautia* and *Clostridium* 38. Furthermore, the abundance of *Streptococcus* was positively correlated with blood urea nitrogen and proteinuria, whereas *Enterobacter* abundance was positively correlated with urinary protein levels 38. These findings suggest that microbial dysbiosis exacerbates renal dysfunction in HN by increasing uremic toxin levels and promoting metabolic disturbances and inflammatory reactions (**Figure 2** and **Figure 4**) 38. In another study, Qiu et al. found that the relative abundances of *Apiotrichum*, *Cystobasidium*, and *Saccharomyces* were increased in HN patients, whereas those of *Bjerkandera*, *Candida*, and *Meyerozyma* were decreased 151. The abundance of *Apiotrichum* and *Saccharomyces* was positively correlated with renal function, while *Saccharomyces*, *Nakaseomyces*, and *Septoria* were associated with diastolic blood pressure 151. Further analysis revealed that microbial dysbiosis influences HN progression by modulating immune responses, elevating pro-inflammatory cytokines and TNF-α levels while decreasing anti-inflammatory cytokine concentrations 151. Yoshifuji et al. demonstrated that compared with sham-operated controls, 5/6 NX rats with hypertension exhibited an increased abundance of *Bacteroides* and a decreased abundance of *Lactobacillus*, which correlated with urinary protein excretion 152. Similarly, animal studies have shown that compared with healthy controls, CKD rats with hypertension displayed decreased abundances of *Ligilactobacillus* and *Ruminococcus* but increased abundances of *Eubacterium* and *Duncaniella* 153. Further evidence indicates that microbial dysbiosis exacerbates renal inflammation and accelerates the progression of HN by impairing intestinal barrier integrity and increasing permeability, thereby facilitating the translocation of harmful metabolites, such as pro-inflammatory cytokines and uremic toxins, into the systemic circulation (**Figure 2**) 152, 153. These findings suggest that dysbiosis of the gut microbiota plays a pivotal role in HN pathogenesis. A comprehensive investigation of its characteristics and mechanisms may provide valuable insights into novel microbiota-targeted therapeutic strategies for the treatment of HN.

#### 3.2.7. LN

As a major form of glomerulonephritis, LN represents one of the most severe organ manifestations of systemic lupus erythematosus (SLE) 37. Recent studies investigating the association between the gut microbiota and LN have revealed the crucial role of the intestinal microbiome in disease progression and therapeutic response, drawing increasing research attention. Patients with SLE exhibit gut microbial dysbiosis characterized by reduced microbial diversity and elevated serum LPS levels 154. As LN reflects renal involvement in SLE, it is hypothesized that patients with LN experience similar microbial alterations 155, 156. Azzouz et al. reported that disease onset in patients is associated with an increased abundance of *Ruminococcus gnavus* 155. Subsequent studies demonstrated that symbiotic bacteria can promote LN progression by disrupting intestinal permeability, amplifying systemic inflammation, and activating the immune system (**Figure 4**) 155, 156. Mu et al. further observed that LN mice displayed a decreased abundance of *Lactobacillales* and increased intestinal permeability 157. Notably, restoration of microbial balance alleviated LN manifestations by reducing B cell infiltration in the kidneys of lupus-prone mice 37. Animal studies have shown that increasing the abundance of beneficial bacteria, such as *L. johnsonii* and *Romboutsia ilealis*, and upregulating plasma tryptophan metabolites activate AHR signaling pathway 158. This activation leads to decreased plasma autoantibody levels, reduced inflammatory cytokine expression, and an overall improvement in LN pathology 158. In summary, microbial dysbiosis contributes to the onset and progression of LN by disrupting the intestinal barrier integrity and promoting systemic immune activation. Targeted interventions aimed at restoring microbial homeostasis and regulating the associated metabolic pathways may represent promising therapeutic strategies for LN management.

#### 3.2.8. PD and HD

Patients with CKD who progress to ESRD require renal replacement therapies, such as PD and HD 159, 160. Dialysis treatment significantly alters gut microbial composition and metabolic profiles 161-163. PD patients exhibited an increased relative abundance of proteolytic bacteria and a decreased abundance of beneficial genera, such as *Lactobacillus* and *Bifidobacterium* 162, 163. These alterations reduce the production of SCFAs while increasing the levels of uremic toxins such as PCS and TMAO 162. In contrast, HD patients displayed an increased relative abundance of Bacteroidetes and serum levels of IS and PCS 163. Additionally, HD patients showed a reduced abundance of beneficial genera, such as *Megamonas*, which is responsible for acetate and propionate production 164. Both dialysis modalities are also associated with disruption of the intestinal barrier integrity, which facilitates the translocation of endotoxins and contributes to systemic inflammation 165. These findings highlight the complex interplay between gut microbiota, metabolic dysregulation, and systemic health in patients undergoing renal replacement therapy.

#### 3.2.9. Kidney transplantation

Kidney transplantation is regarded as the optimal treatment for ESRD, providing superior survival and quality of life compared with dialysis 10, 166. However, alterations in the gut microbiota and metabolite profiles have been observed in kidney transplant recipients, particularly in those experiencing antibody-mediated renal allograft rejection or post-transplant diarrhea 167, 168. Recipients with antibody-mediated rejection exhibit reduced gut microbial richness and diversity, accompanied by significant changes in key metabolites, such as 3β-hydroxy-5-cholic acid and taurocholic acid, both of which correlate strongly with microbial compositional shifts 168. Furthermore, transplant recipients with post-transplant diarrhea display decreased abundances of beneficial bacteria such as *Eubacterium* and *Ruminococcus*, along with increased abundances of pro-inflammatory taxa such as *Enterococcus* and *Escherichia* 167. These microbial changes are associated with alterations in carbohydrate and amino acid metabolism, suggesting that diarrhea may result from a dysbiotic state 167. Together, these findings emphasize the intricate relationship between gut microbiota dysbiosis and metabolic dysfunction in kidney transplant recipients, highlighting the need for microbiome-targeted strategies to improve post-transplant outcomes.

### 3.3 The transition from AKI to CKD

For patients with AKI, recovery of renal function is a critical determinant of survival and long-term prognosis 169. Although many patients experience reversible improvement, a substantial proportion continue to exhibit progressive renal decline, ultimately leading to CKD 169. With increasing recognition of the gut-kidney axis, recent studies have highlighted the pivotal role of gut microbial dysbiosis in the transition from AKI to CKD 169, 170. For example, in elderly individuals, reduced microbial diversity and decreased abundance of beneficial bacteria lead to lower SCFA levels and increased intestinal permeability 170. These changes promote low-grade chronic inflammation, thereby facilitating the progression of AKI to CKD 170. Animal studies have shown that compared with age-matched sham mice, IRI mice exhibit reduced abundance of *Lactobacillus* and *Bifidobacterium* 171. Concurrently, microbial dysbiosis contributes to persistently elevated levels of inflammatory cytokines during the AKI-to-CKD transition 171. Zhu et al. reported that IRI-induced AKI mice with gut dysbiosis exhibited reduced SCFA levels and impaired metabolic activity in the renal tubular epithelial cells, which collectively promoted CKD development and renal fibrosis 169. Similarly, Lee et al. observed that compared with the sham group, mice undergoing the AKI-to-CKD transition displayed decreased abundance of Actinobacteria and Firmicutes 169. Moreover, gut microbial alterations, such as enrichment of *Enterorhabdus*, are associated with elevated plasma TMAO levels, leading to exacerbated renal fibrosis and functional decline through activation of oxidative stress and inflammatory pathways (**Figure 4**) 169. These findings demonstrate the pivotal role of gut microbiota in mediating the transition from AKI to CKD. Further research into the mechanistic interactions between gut dysbiosis, inflammation, and metabolic reprogramming is essential for understanding kidney disease progression and identifying effective microbiota-targeted therapeutic strategies.

## 4. Targeting gut microbiota as a promising therapy for the treatment of kidney disease

Numerous studies have demonstrated that gut microbial dysbiosis is intricately involved in the development and progression of kidney disease (**Figure 2**) 33, 39, 172. Consequently, targeting gut microbiota has emerged as a promising therapeutic strategy for treating kidney diseases. Increasing evidence indicates that both AKI and CKD can be ameliorated through several microbiota-directed interventions, including microbial therapeutics (probiotics, prebiotics, and synbiotics), natural products (traditional Chinese medicines (TCM) and natural compounds), and FMT (**Figure 2** and **Table 2**) 81, 84, 173.

### 4.1 Reshaping gut microbiota in kidney disease by gut microecological prescription

#### 4.1.1 Remodeling gut microbiota in AKI by microbial therapeutics

The modulation of intestinal microbiota through probiotics, prebiotics, and symbiotic bacteria has emerged as a potential therapeutic approach for managing AKI 170, 174. A growing body of evidence highlights the importance of maintaining intestinal barrier integrity to prevent the translocation of harmful bacteria and their metabolites into systemic circulation 102. Probiotics-defined as live microorganisms that confer health benefits on the host-have shown efficacy in protecting against AKI 170, 174. For instance, *Lactobacillus casei Zhang* (*L. casei Zhang*) and *P. goldsteinii* (**Table 2**) modulate gut microbiota-derived metabolites and prevent AKI by regulating immune and inflammatory responses (**Figure 3**) 100, 170. Furthermore, *Pediococcus acidilactici GKA4*, its heat-killed form (“death probiotic” GKA4), and postbiotic GKA4 have been shown to mitigate cisplatin-induced AKI by reducing serum biomarkers and renal histopathological injury (**Table 2**) 100, 174. These findings highlight the intricate relationship between intestinal health and renal function, suggesting that microbiome modulation can improve the clinical outcomes of patients with AKI 175. Consequently, targeted combinations of symbiotic bacteria may enhance the intestinal microbial composition, attenuate renal tissue injury, and improve kidney function indicators in AKI.

#### 4.1.2 Reshaping gut microbiota in CKD by microbial therapeutics

Dietary interventions aimed at increasing the production of SCFAs have gained prominence because of the strong correlation between CKD and microbial dysbiosis 176. Probiotics, prebiotics, and synbiotics exert renoprotective effects by modulating the gut-kidney axis, thereby alleviating uremic toxicity and systemic inflammation (**Figure 3**) 24, 177. These agents promote the proliferation of beneficial bacteria, while suppressing harmful strains (**Table 2**) 178, 179. Clinical studies have demonstrated that probiotic supplementation reduces plasma uremic toxin levels in patients with CKD by increasing beneficial bacteria and decreasing harmful metabolites associated with dysbiosis 180, 181. For example, supplementation attenuated renal injury by inhibiting AHR pathway through its metabolite IAld in adenine-induced CKD rats (**Figure 3**) 106. Similarly, animal studies have shown that probiotics, such as *F. prausnitzii*, *B. ovatus,* and *B. fragilis*, inhibit renal inflammation and fibrosis via multiple molecular mechanisms (**Table 2**) 107-109. These include modulation of butyrate-renal GPR43 expression, promotion of intestinal hyodeoxycholic acid synthesis through upregulation of *C. scindens*, and reduction of LPS while increasing 1,5-anhydroglucitol levels (**Figure 3**) 107-109. In addition, treatment with *Bifidobacterium animalis* A6 has been shown to ameliorate renal fibrosis and glomerulosclerosis by decreasing the relative abundance of toxin-producing species, such as *Eggerthella lenta* and *Fusobacterium*, and by reducing serum levels of uremic toxins, creatinine, and urea in CKD rats 110.

Synbiotic supplementation has also been reported to beneficially modulate inflammation, oxidative stress, and lipid metabolism, with evidence demonstrating its efficacy in lowering IS and PCS levels 182, 183. These effects are mediated through restoration of gut microbial balance and repair of intestinal barrier integrity, thereby reducing the uremic toxin burden and alleviating cardiovascular and metabolic disorders 184. Animal experiments further support the potential of synbiotics to improve gut microbial composition, reduce uremic toxins, and prevent renal function decline in CKD models 183, 185. In summary, probiotics, prebiotics, and synbiotics represent promising therapeutic approaches for CKD management, offering mechanistically grounded interventions targeting the gut-kidney axis.

#### 4.1.3 Regulating gut microbiota in the transition from AKI to CKD by microbial therapeutics

Recently, increasing attention has been directed toward the use of probiotics to modulate disease progression during the transition from AKI to CKD. For example, Zhu et al. reported that the probiotic *L. casei Zhang* slowed the progression of both AKI and CKD by modulating inflammatory responses in renal tubular epithelial cells and macrophages via gut microbiota-derived metabolites 170. Animal studies further demonstrated that *L. casei Zhang* not only enhanced intestinal mucosal barrier integrity and suppressed inflammatory signaling but also exerted renoprotective effects through mechanisms independent of the native microbiota 170. Kim et al. found that in IRI-induced aged mice, probiotic supplementation with *Bifidobacterium bifidum* and *Bifidobacterium longum* reduced the abundance of *Parabacteroides* and *Akkermansia*, while increasing the abundance of *Alistipes*, Muribaculaceae, and Oscillospiraceae 171. Additional animal studies have confirmed that ameliorating microbial dysbiosis delays the AKI-to-CKD transition by elevating SCFA levels, improving intestinal barrier function, and modulating inflammatory responses 171. Furthermore, *L. casei Zhang* mitigated AKI and subsequent chronic renal fibrosis by increasing SCFA and nicotinamide levels and restoring gut microbial homeostasis 186. In summary, the targeted modulation of microbial dysbiosis during the AKI-to-CKD transition represents an effective strategy to prevent CKD development by enhancing intestinal integrity, reducing inflammation, and promoting metabolic homeostasis.

### 4.2 Reshaping gut microbiota in kidney disease through natural products

Natural products, including TCMs and plant extracts, have long been used and are widely recognized as important therapeutic interventions for diverse kidney diseases, including AKI and CKD 132, 187, 188. Recent studies have highlighted the ability of TCMs to modulate the intestinal microbiota and restore metabolic homeostasis in both AKI and CKD (**Table 3**) 96, 189-191.

#### 4.2.1 Regulating gut microbiota in AKI through natural products

Accumulating evidence demonstrates that natural products can ameliorate AKI by modulating gut microbiota (**Table 3**) 96, 97. Zou et al. reported that Qiong-Yu-Gao (QYG), a TCM formula derived from *Poria*, *Ginseng Radix*, and *Rehmanniae Radix*, protects against cisplatin-induced AKI by enhancing the fecal abundance of *Akkermansia*, *Faecalibaculum*, and *Bifidobacterium* 96. These microbial changes are associated with altered metabolite profiles, including increased acetate and butyrate levels, reduced IS and PCS concentrations, and improvements in AKI biomarkers and indicators of fibrosis and inflammation 96. Mechanistically, QYG treatment inhibited histone deacetylase expression and activity 96. The renoprotective effects were abolished following antibiotic-induced microbial depletion but were transferable via FMT, confirming a microbiota-dependent mechanism (**Figure 5**) 96. Yang et al. demonstrated that *Aconitum carmichaelii* Debx attenuates cisplatin-induced AKI by restoring microbial homeostasis, increasing SCFA levels, and decreasing uremic toxins 97. This restoration was accompanied by the recovery of glutathione and tryptophan metabolism, inhibition of IκB/NF-κB signaling, and activation of Keap1/Nrf2 signaling pathways (**Figure 5**) 97. Zhu et al. reported that chromone hamaudol, an oxygen-containing heterocyclic compound, ameliorated AKI-associated renal injury by enhancing the abundance of *P. goldsteinii* 99. Additionally, *Flammulina velutipes* polysaccharides (FVP) reduced the abundance of Proteobacteria, *Enterococcus*, and S*higella* while increasing the abundance of Firmicutes, *Lactobacillus*, *Ruminococcus*, *Bifidobacterium*, *Lactococcus*, *Christensenella*, and *Allobaculum* 192. These microbial shifts were accompanied by elevated SCFA levels, particularly acetate and butyrate 192. Kidney metabolomic analysis revealed that FVP treatment inhibited ferroptosis by increasing glutathione (GSH), glutathione peroxidase 4 (GPX4), and solute carrier family 7 member 11 (SLC7A11) expression while reducing AA accumulation (**Figure 5**) 192. These findings illustrate the potential of TCMs and natural products to beneficially modulate the gut-kidney axis, thereby alleviating AKI and potentially preventing its progression to CKD (**Figure 5**).

#### 4.2.2 Regulating gut microbiota in CKD through natural products

Compared with AKI, a larger body of research has reported that natural products ameliorate CKD by reshaping microbial dysbiosis (**Table 3**) 189-191, 193. Liu et al. found that Zicuiyin decoction reduced serum creatinine levels in patients with DKD, accompanied by an increased abundance of Prevotellaceae and Lactobacillaceae and a decreased abundance of Enterobacteriales, Clostridiaceae, and Micrococcaceae (**Table 3**) 189. Dong et al. reported that Yi-Shen-Hua-Shi granules, a traditional Chinese patent medicine used to treat patients with CKD, increased the relative abundance of Lachnospiraceae, *Sutterella*, *Lachnoclostridium*, and *Faecalibacterium*, while reducing *Eggerthella* and *Clostridium innocuum* group after four months of treatment 190. Reduction in proteinuria was positively correlated with the abundance of Lachnospiraceae and *Lachnoclostridium*, suggesting a link with vitamin, lipid, and glycan metabolic pathways (**Table 3**) 190. Lin et al. further demonstrated that Fushen granules increased the abundance of *Bacteroides*, *Rothia*, and *Megamonas*, which was correlated with alterations in amino acid and carbohydrate metabolism 193. These findings indicate that multi-herbal TCM preparations improve renal function in patients with CKD by modulating gut microbiota composition and metabolism.

An expanding number of studies have also emphasized that natural compounds serve as valuable sources for new drug development 194-197. Our research group demonstrated that treatment with both barleriside A and 5,6,7,8,3',4'-hexamethoxyflavone improved renal function and fibrosis while increasing *L. johnsonii* abundance in CKD rats (**Table 3**) 106, 198, 199. Liu et al. reported that madecassoside, an oxolane-type triterpene glycoside, improves renal function and attenuates fibrosis, while promoting the growth of *B. fragilis* (**Table 3** and** Figure 5**) 109. The same group also showed that neohesperidin decreased serum urea and creatinine levels, ameliorated renal fibrosis, and increased *B. ovatus* abundance in mice with adenine-induced CKD and UUO (**Table 3** and **Figure 5**) 108. These findings suggest that natural compounds alleviate renal fibrosis through gut microbiome-dependent pathways. Wang et al. further demonstrated that isoquercitrin modulates the microbial electron transport chain, thereby regulating tryptophan transport and indole biosynthesis in adenine-induced CKD mice (**Table 3** and** Figure 5**) 200. In addition, treatment with coumarins isolated from *Hydrangea paniculata* improved colonic integrity, decreased the Firmicutes-to-Bacteroidetes ratio, reduced the relative abundance of *Allobaculum* and *Desulfovibrio*, decreased uremic toxin precursors, and enhanced SCFA production in MN rats 136. Taken together, these studies demonstrate that natural compounds hold great promise as therapeutic agents for kidney diseases (**Figure 5**).

### 4.3 Reshaping gut microbiota in kidney disease through FMT

#### 4.3.1 Remodeling gut microbiota in AKI through FMT

FMT represents a novel and promising strategy for reshaping intestinal microbiota in AKI 173. In experimental models, FMT has been shown to alleviate renal lesions, improve renal function, and reduce inflammatory responses in experimental models 201-203. Moreover, FMT mitigates the adverse effects of chemotherapeutic nephrotoxicity by restoring intestinal microbial balance and promoting kidney recovery following AKI 203. These findings suggest that FMT is a promising adjunct therapy for AKI.

#### 4.3.2 Reshaping gut microbiota in CKD through FMT

FMT has also emerged as a promising therapeutic approach for restoring intestinal homeostasis in CKD patients 204-206. Clinical and preclinical studies have demonstrated that FMT effectively restores microbial diversity and composition, thereby ameliorating microbial dysbiosis 173. A clinical trial further reported that FMT facilitates the decolonization of multidrug-resistant organisms by replacing resistant strains with beneficial non-extended-spectrum beta-lactamase bacteria, highlighting a potential role for FMT in combating antimicrobial resistance 207. In addition, FMT has been shown to reduce proteinuria and gastrointestinal symptoms in patients with IgAN and MN, while simultaneously enhancing the production of beneficial metabolites such as SCFAs and reducing levels of harmful uremic toxins 204, 206. Furthermore, FMT modulates immune responses by counteracting CKD-associated inflammation and metabolic dysfunction 208. Preclinical studies have demonstrated that FMT benefits DKD models by restoring gut microbiota composition, enhancing microbial diversity, and reducing tubulointerstitial injury and inflammatory cytokines 209. The study also emphasized the critical role of acetate-producing gut flora and their metabolites in mediating the metabolic and inflammatory balance 209.

Additional evidence indicates that FMT effectively mitigates microbial dysbiosis and reduces circulating uremic toxins, including p-cresyl glucuronide and PCS, which are strongly associated with CKD progression 210. Taken together, these findings support FMT as an emerging therapeutic modality that not only restores microbial equilibrium but may also slow CKD progression through multi-target modulation of metabolic, inflammatory, and immune pathways.

## 5. Conclusion and challenges

Growing evidence supports a reciprocal relationship between the host and the intestinal microbiome across various kidney diseases. Further studies are critically needed to delineate the distinct features of the gut microbiome in kidney disorders and clarify its specific associations with different renal pathologies. Intestinal inflammation and compromised epithelial barrier integrity accelerate the systemic dissemination of bacterially derived uremic toxins such as IS, PCS, and TMAO, which induce oxidative stress and cause progressive renal damage. Recent research on the gut-kidney axis has revealed novel therapeutic pathways to alleviate inflammation, renal injury, and uremia, thereby preventing adverse clinical outcomes in CKD (**Figure 2**). Multiple promising strategies have been proposed to restore gut microbial equilibrium and slow the progression of kidney diseases. Approaches such as gut microecological prescriptions and TCM formulations provide innovative, signaling-directed interventions that may surpass conventional pharmacotherapies, often burdened by adverse effects. Moreover, the selection of probiotic strains with well-defined metabolic and immunomodulatory functions has the potential to mitigate diverse renal pathologies (**Figure 2**). Future studies should rigorously evaluate these interventions in large-scale clinical trials to translate microbiome-based strategies into tangible clinical benefits for CKD patients. Mounting evidence has identified key microorganisms, microbial enzymes, and metabolites as potential therapeutic targets. An enhanced grasp of host-microbiome metabolic interactions will pave the way for developing innovative probiotics, prebiotics, and synbiotics, facilitating the emergence of personalized, microbiota-targeted therapies for preventing and treating kidney diseases.

## Figures and Tables

**Table 1:** The effects of microbial-derived metabolites on kidney disease

Metabolites	Microbial source	Disease models	Molecular mechanism	Ref.
IS	*C. sporogenes*,* C. bartlettii*,* B. longum*,* B. fragilis, P. distasonis*,* E. hallii*	IS-induced mice, monocyte-derived macrophages, and IEC-6 Cells	Mediating renal fibrosis and inflammation by activating AHR and NF-κB pathways.	39, 84
PCS	*Adlercreutzia*, *unclassified Erysipelotrichaceae*	NX rats and PCS-induced HK-2 cells	Mediating renal fibrosis and inflammatory cytokines by activating protein kinase C and PI3K signaling pathways.	39, 84
TMAO	*Lachnospiraceae_UCG-002*	CKD and TMAO-induced mice, NX rats, and HK-2 cells	Inducing renal dysfunction and fibrosis by activating AHR/p38MAPK/NF-κB pathway and NLRP3 inflammasome.	39, 84
IAA	*C. bartlettii*,* C. scatologenes*,* C. drakei*, *E. cylindroides*,* E. rectale*, *B. thetaiotaomicron*, *B. fibrisolvens*, *M. hypermegale*, *P. distasonis*	CKD patients and IAA-induced endothelial cells	Aggravating renal fibrosis and inflammation by activating AHR, p38MAPK, and NF-κB pathways.	39, 84
Acetate	*Akkermansia muciniphila*,* Blautia hydrogenotrophica*	DKD mice, CKD mice, and glomerular mesangial cells	Improving renal fibrosis by reducing the expression of the fibrosis-related genes TGF-β and fibronectin	89
Propionate	*Phascolarctobacterium succinatutens*,* Megasphera elsdenii*,* C. catus*,* Ruminococcus obeum*, *Roseburia inulinivorans*	DKD mice, CKD mice, and podocytes	Attenuating renal inflammation by inhibiting oxidative stress through reducing ROS, NF-κB activation, and the release of MCP-1 and IL-1β.	89
Butyrate	*F. prausnitzii*,* C. viene*, *C. eutacus*, *C. catus*, *E. rectale*, *E. hallii*, *F. prausnitzii*	ESRD patients, DKD mice, and HK-2 cells	Attenuating inflammation and renal fibrosis by inhibiting ERK/MAP and NF-κB pathways.	89, 110
IAld	*L. johnsonii*	CKD patients, NX and UUO rats, and 1-hydroxypyrene-mediated HK-2 cells	Improving renal function and fibrosis by ameliorating AHR pathway.	89, 110
HDCA	*B. ovatus*, *C. scindens*	UUO and adenine-induced CKD mice	Attenuating renal fibrosis by inhibiting TGF-β/Smad3 and ERK1/2 pathways.	89, 110
1,5-AG	*B. fragilis*	UUO and adenine-induced CKD mice	Improving renal fibrosis by increasing SGLT2 and inhibiting TGF-β/Smad pathway.	106

**Table 2:** The effects of gut microecological prescription on gut microbiota, metabolites, and molecular mechanisms in AKI and CKD

Intervention method	Patients/animal models	Dose	Microbial dysbiosis	Metabolic pathways	Molecular mechanisms	Ref.
*P. goldsteinii*	IRI-induced AKI mice	1×10^8^ CFU	Increasing *P. goldsteinii*.	Decreasing LPS level.	Blunting renal injury by reducing inflammation, cell apoptosis, and ROS accumulation.	100
*Pediococcus acidilactici* GKA4, etc.	Cisplatin-induced AKI mice	250 mg/kg	Increasing* Pediococcus acidilactici*, etc.	Reducing malondialdehyde level, increasing glutathione level.	Alleviating AKI by enhancing activating HO-1/Nrf2 pathway, and mitigating apoptosis and autophagy pathway.	174
*L. casei* Zhang	CKD patients, bilateral I/R-induced AKI and CKD mice	1×10^9^ CFU	Altering *Alloprevotella, NK3B31* groups, *Allobaculum*, etc.	Increasing SCFA and nicotinamide levels.	Improving microbial dysbiosis, intestinal inflammation, promoting kidney repair, and alleviating chronic TIF.	170
*L. johnsonii*	Denine-induced CRF rats	0.2 µL of 1×10^9^ CFU	Increasing *L. johnsonii*	Increasing IAld levels.	Ameliorating renal fibrosis by restoring microbial dysbiosis and inhibiting AHR pathway via increasing IAld level.	106
*F. Prausnitzii*	5/6 nephrotomy-induced CKD mice	200 µL of 2×10^8^ CFU	Altering *Roseburi*, *Faecalibacterium*, *Collinsella*, etc.	Increasing butyrate levels, reducing PCS and TMAO levels.	Alleviating renal fibrosis and inflammation by reducing intestinal inflammation and permeability, and upregulating GPR43 expression.	107
*B. ovatus*	UUO and adenine-induced CKD mice	200 µL of 2×10^8^ CFU	Increasing *B. ovatus* and *C. scindens*.	Increasing gut HDCA level.	Attenuating renal fibrosis by raising HDCA level, reducing LPS absorption, and inhibiting TGF-β/Smad3 and ERK1/2 pathways.	108
*B. fragilis*	UUO and adenine-induced CKD mice	200 µL of 2×10^8^ CFU	Increasing *B. fragilis.*	Increasing 1,5-AG level, decreasing LPS level.	Improving renal fibrosis by reducing LPS level, increasing SGLT2, and inhibiting TGF-β/Smad pathway.	109

**Table 3:** The effects of natural products on gut microbiota, metabolites, and molecular mechanisms in AKI and CKD

Intervention method	Patients/animal model	Dose	Microbial dysbiosis	Metabolic pathways	Molecular mechanisms	Ref.
*Aconitum carmichaelii* Debx	Cisplatin-induced AKI mice	5, 10 mg/kg	Altering *Ligilactobacillus*, *Bacteroides*, *Escherichia-Shigella*, etc.	Increasing SCFA levels, decreasing uremic toxins, and kynurenic acid levels.	Alleviated AKI by inhibiting NF-κB and promoting Nrf2/HO-1 signalling pathways.	97
FVP	Cisplatin-induced AKI mice	50, 100, 200 mg/kg	Increasing *Lactobacillus*, *Bifidobacterium*,* Enterococcus*, *Shigella*, etc.	Increasing SCFA level, decreasing AA level.	Retarding renal fibrosis by inhibiting ferroptosis via increasing GSH, GPX4, and SLC7A11 expression.	192
Hamaudol	IRI-induced AKI mice	30 mg/kg	Increasing *P. goldsteinii*	Decreasing LPS level.	Improving renal injury by retarding intestinal barrier, renal inflammation, and cell apoptosis.	100
Qiong-Yu-Gao	Cisplatin-induced AKI mice	7 g/kg	Increasing *Faecalibaculum* and *Bifidobacterium*, decreasing *Lactobacillus*, and* Bacteroides*	Increasing SCFA level, reducing uremic toxin level.	Improved renal fibrosis by suppressing histone deacetylase expression and activity.	96
Zicuiyin decoction	DKD patients	75 g/100 ml	Altering *Lactobacillaceae*, *Enterobacteriales*,* Clostridiaceae*, etc.	Increasing SCFA level, decreasing creatinine level.	Improving kidney function by ameliorating gut microbiota and relieving persistent albuminuria.	189
Yi-Shen-Hua-Shi Granules	CKD patients	3 bags/day	Altering Bacteroides,* Sutterella*, *Clostridium innocuum group*, etc.	Increasing SCFA level.	Mitigating CKD by regulating metabolism of polysaccharides, lipids, and vitamins via altering gut microbiota.	190
Suyin Detoxification granules	CKD rats	3.3, 6.6 g/kg	Increasing Lactobacillus, *Bacteroides*, and *Rikenellaceae_RC9_gut_group*	Decreasing serum TMAO level.	Mitigating CKD by affecting lipid metabolism and renal tubular ferroptosis.	211
Yiqi-Huoxue-Jiangzhuo formula	NX mice	23.6, 11.8, 5.9 g/kg	Increasing Lachnospiraceae-*NK4A136-group* but decreasing *Eubacterium-nodatum-group*	Decreasing serum TMAO level	Mitigated renal injury and exerted cardiac protective effects by inhibiting NLRP3 inflammasome.	212
BSA and HMF	Adenine-induced CKD rats	10 mg/kg	Increasing* L. johnsonii*	Increasing IAld level.	Improved renal fibrosis by inhibiting profibrotic protein expression.	106
Neohesperidin	UUO and adenine-induced CKD mice	50 mg/kg	Increasing* B. ovatus*	Increasing HDCA level.	Retarding renal fibrosis by increasing *Bacteroides ovatus*.	108
Madecassoside	UUO and adenine-induced CKD mice	80 mg/kg	Increasing* B. fragilis*	Increasing 1,5-AG level.	Retarding renal fibrosis by increasing *B. fragilis*, inhibiting oxidative stress, and TGF-β/Smad pathway.	109
Isoquercitrin	Adenine-induced CKD mice	80 mg/kg	Decreasing* E. coli*	Decreasing IS level.	Blunting CKD by inhibiting tryptophan transport and indole synthesis via gut bacteria.	200
*Moutan cortex* polysaccharides	high-sugar diet and STZ-induced DKD rats	80, 160 mg/kg	Altering *Akkermansia*, *Ruminococcus*, *Ruminococcus_2*, etc.	Increasing SCFA level but decreasing branched-chain fatty acids and LPS levels.	Mitigating renal injury by reducing pro-inflammatory mediators and increasing SCFA.	213
Paotianxiong polysaccharides	Adenine-induced CKD rats	62.5, 125, 250 mg/kg	Altering *B. fragilis*, *L. murinus*, *L. animalis*, and* P. aeruginosa*	Decreasing stearic acid, linoleic acid, and docosahexaenoic acid levels.	Improving kidney damage by regulating unsaturated fatty acid metabolism and reducing inflammatory factors.	214
*Astragalus membranaceus* and *Salvia miltiorrhiza*	DKD rats	2.95, 5.9 g/kg	Altering *Akkermansia_muciniphila*, *Lactobacillus*, *Lactobacillus_murinus*	Decreasing IS and PCS levels.	improving DKD by inhibiting inflammation and regulating glucose and lipid metabolism.	127
